# Enhanced Generation of Induced Pluripotent Stem Cells from a Subpopulation of Human Fibroblasts

**DOI:** 10.1371/journal.pone.0007118

**Published:** 2009-09-23

**Authors:** James A. Byrne, Ha Nam Nguyen, Renee A. Reijo Pera

**Affiliations:** Center for Human Embryonic Stem Cell Research and Education, Institute for Stem Cell Biology and Regenerative Medicine, Department of Obstetrics and Gynecology, Stanford University, Palo Alto, California, United States of America; Johns Hopkins University, United States of America

## Abstract

**Background:**

The derivation of induced pluripotent stem cells (iPSCs) provides new possibilities for basic research and novel cell-based therapies. Limitations, however, include our current lack of understanding regarding the underlying mechanisms and the inefficiency of reprogramming.

**Methodology/Principal Findings:**

Here, we report identification and isolation of a subpopulation of human dermal fibroblasts that express the pluripotency marker stage specific embryonic antigen 3 (SSEA3). Fibroblasts that expressed SSEA3 demonstrated an enhanced iPSC generation efficiency, while no iPSC derivation was obtained from the fibroblasts that did not express SSEA3. Transcriptional analysis revealed NANOG expression was significantly increased in the SSEA3 expressing fibroblasts, suggesting a possible mechanistic explanation for the differential reprogramming.

**Conclusions/Significance:**

To our knowledge, this study is the first to identify a pluripotency marker in a heterogeneous population of human dermal fibroblasts, to isolate a subpopulation of cells that have a significantly increased propensity to reprogram to pluripotency and to identify a possible mechanism to explain this differential reprogramming. This discovery provides a method to significantly increase the efficiency of reprogramming, enhancing the feasibility of the potential applications based on this technology, and a tool for basic research studies to understand the underlying reprogramming mechanisms.

## Introduction

The generation of pluripotent stem cells that are genetically identical to an individual provides unique opportunities for basic research and for potential immunologically-compatible novel cell-based therapies [1]. Methods to reprogram primate somatic cells to a pluripotent state include somatic cell nuclear transfer [2], somatic cell fusion with pluripotent stem cells [3] and direct reprogramming to produce induced pluripotent stem cells (iPSCs) [4]–[10]. These methodologies, however, are characterized by a low reprogramming efficiency and a lack of knowledge regarding the underlying mechanisms. While it has been demonstrated previously that more differentiated cells demonstrate a lower reprogramming efficiency [11] and different somatic cell types possess differential reprogramming ability [12], [13], no study to date, to our knowledge, has identified subpopulations of cells within a primary cell population possessing differential reprogramming potential. If such subpopulations exist and can be identified and isolated, they provide a method to significantly increase the efficiency of reprogramming, enhancing the feasibility of the potential applications based on this technology [1], and a tool for basic research studies to understand the underlying reprogramming mechanisms.

## Results

**Figure 1:**
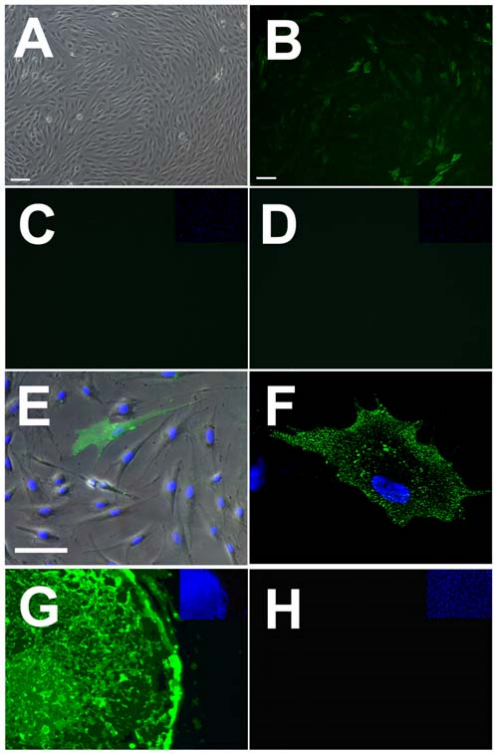
Expression of SSEA3 from primary human dermal fibroblasts. (A–B) Primary adult human fibroblast line HUF1 (A) Phase contrast image (B) Immunocytochemical detection of SSEA3 expression (green). (C–D) Immunofluorescence staining for (C) TRA-1-60 and (D) TRA-1-81 on HUF1 cells. (E) Overlay of SSEA3 expression on phase contrast image of HUF1 cells. (F) Confocal section through primary human fibroblast (HUF1) cell demonstrating SSEA3/488 detection primarily from the cell membrane in addition to localized peri-nuclear detection. (G) SSEA3/488 detection on H9 human embryonic stem cells. (H) 488 secondary antibody only negative control staining of HUF1 cells. (C–H) DAPI staining in blue. Scale bars represent 100 microns.

We derived a fibroblast line from a skin biopsy from a healthy adult male (HUF1) (Figure 1A) and used immunocytochemistry to characterize the expression of cell surface markers commonly expressed on pluripotent stem cells (Figure 1B, C and D). Unexpectedly, we observed that, even prior to reprogramming, the HUF1 line possessed cells that demonstrated heterogeneous expression of stage specific embryonic antigen 3 (SSEA3; Figure 1B). SSEA3 is a cell surface glycosphingolipid considered an embryonic/pluripotency marker [14], [15]. Overlaying phase contrast and SSEA3 immunofluorescence images revealed that the SSEA3 expression was detected across the entire cell surface (Figure 1E) and using confocal microscopy we observed that the SSEA3 expression was primarily localized to the cellular membrane (Figure 1F). Additional small and localized regions of SSEA3 fluorescence were also detected around the peri-nuclear region, possibly reflecting the intracellular processing and packaging of SSEA3 on peri-nuclear endoplasmic reticulum and/or Golgi bodies (Figure 1F). Notably, in positive controls, strong cell surface expression of SSEA3 was observed in H9 human embryonic stem cells (hESCs)(Figure 1G) and no expression was observed in the negative controls (Figure 1H).

**Figure 2:**
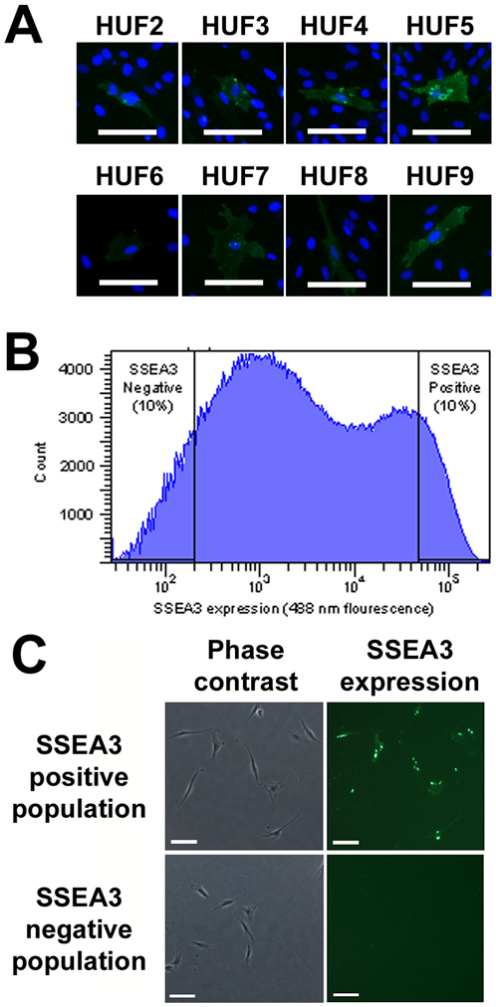
FACS analysis and isolation of SSEA3-positive and SSEA3-negative primary adult human fibroblasts. (A) Immunocytochemical analysis for SSEA3 expression in eight additional primary adult dermal human fibroblast (HUF) lines. DAPI staining in blue. (B) Histogram of FACS analyzed HUF1 cells following live binding of SSEA3/488 antibody complex and gating for SSEA3-positive (top 10%) and SSEA3-negative (bottom 10%) populations. (C) Detection of SSEA3/488 fluorescence signal in FACS sorted SSEA3-positive and SSEA3-negative populations following overnight adherence. (A & C) SSEA3 staining in green. Scale bars represent 100 microns.

We next examined whether the expression of SSEA3 in a subset of fibroblasts was specific to HUF1 or a more general observation. Eight additional primary adult human fibroblast lines were derived from skin biopsies and immunoassayed. We observed that all eight lines contained a subpopulation of cells that were positive for SSEA3 (Figure 2A). Fluorescence activated cell sorting (FACS) analysis of HUF1 cells stained with the SSEA3/488 antibody complex, revealed a larger subpopulation of cells with little or no SSEA3 expression and a smaller subpopulation with detectable SSEA3 expression (Figure 2B). Subsequently, we isolated (through FACS) and cultured the top 10% and bottom 10% of the SSEA3/488 fluorescing cells as our SSEA3-positive and negative populations respectively (Figure 2B). Immunofluorescence analysis of the two populations, following overnight adherence to exclude analysis of non-viable cells, revealed that >97% of the SSEA3-positive population expressed detectable SSEA3/488 fluorescence and 0% of the SSEA3-negative population expressed detectable SSEA3/488 fluorescence (Figure 2C), demonstrating that the fluorescence activated cell sorting process can purify viable subpopulations of cells from a heterogeneous somatic population. These subpopulations were then used for reprogramming to iPSCs.

**Figure 3:**
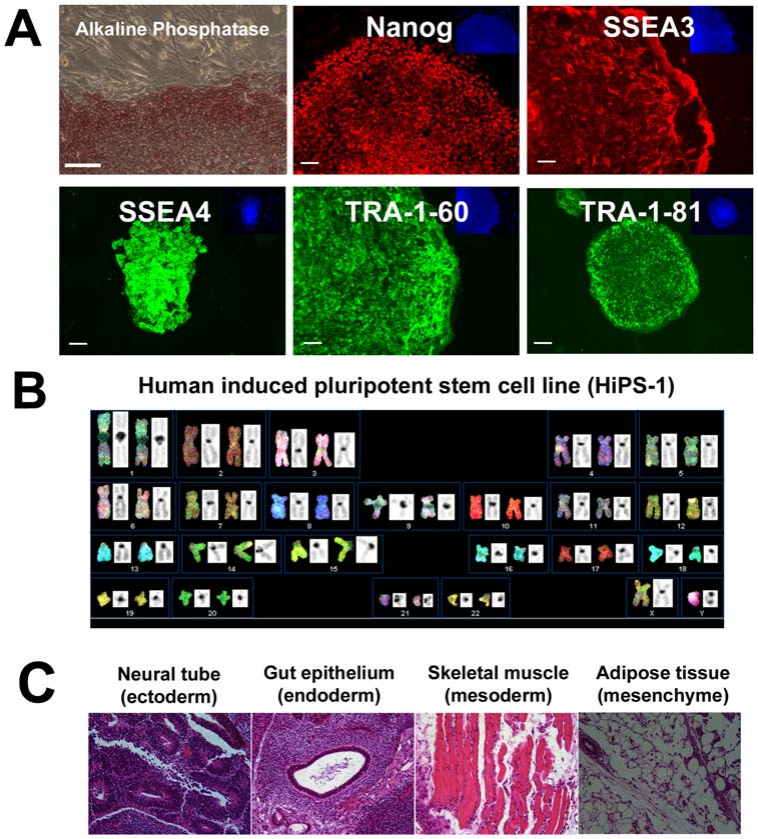
Characterization of unsorted HUF1 derived induced pluripotent stem cells (HiPS-1 control). (A) Expression of pluripotency markers from iPSCs (HiPSC-1 control) generated following retroviral transduction of unsorted HUF1 cells. DAPI staining in blue. Scale bar represents 100 microns. (B) SKY karyotype analysis of the HiPS-1 control line. (C) Histological analysis of teratoma derived from HiPS-1 control line.

**Figure 4:**
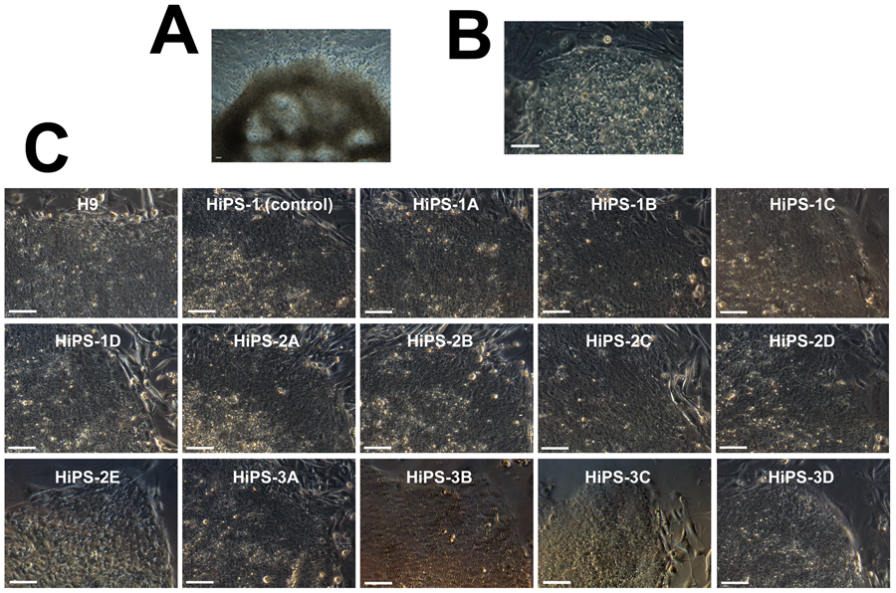
Morphology of colonies and lines following retroviral transduction of HUF1 cells. (A) Large background colony with no ESC-like attributes. (B) ESC-like iPSC colony. (C) Morphology of SSEA3-selected lines following derivation. (A–C) Scale bar represents 100 microns.

**Figure 5:**
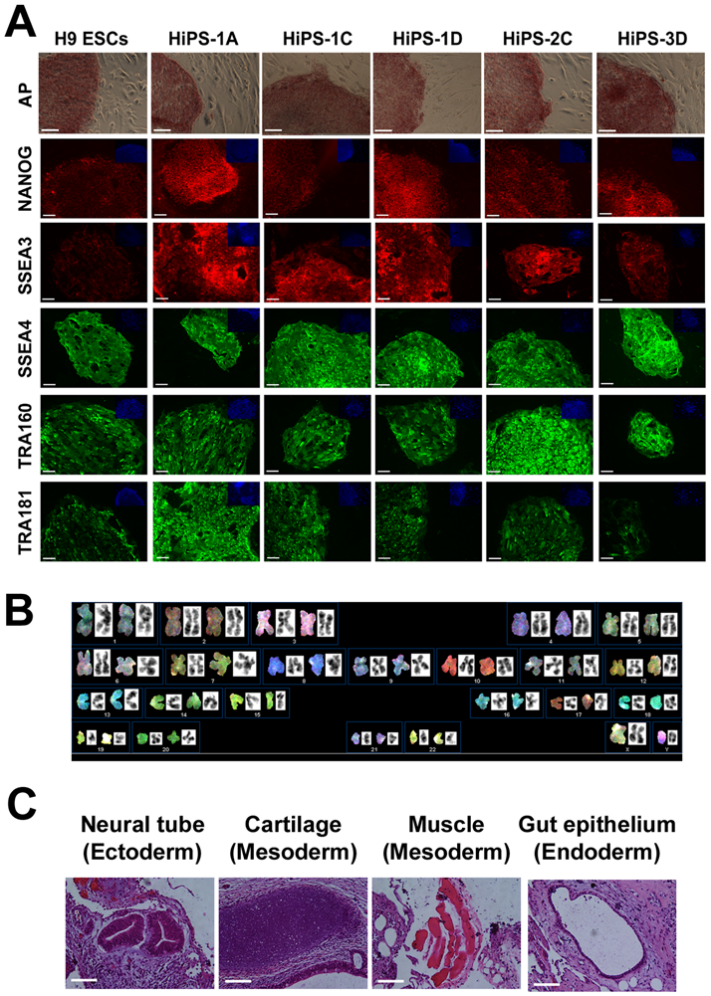
Characterization of SSEA3-selected HiPSCs. (A) Expression of pluripotency markers on H9 ESCs and SSEA3-selected HiPSC lines. Alkaline phosphatase (AP) staining in dark red/purple. DAPI stained images are inset in blue. (B) Spectral karyotype (SKY) of SSEA3-selected HiPS-2C line. (C) Histological analysis of teratoma derived from SSEA3-selected HiPS-2C line. (A & C) Scale bar represents 100 microns.

Previous reprogramming work demonstrated that we could reprogram the entire, unsorted population of HUF1 somatic cells using retroviral vectors that express OCT4, SOX2, KLF4 and cMYC to generate iPSCs that express the same pluripotency markers as control H9 ESCs (Figure 3A). Reprogrammed cells possessed a normal karyotype (Figure 3B), differentiated into beating cardiomyocytes *in vitro* (Movie S1) and differentiated into representatives of all three germ layers *in vivo* (Figure 3C). We transduced our SSEA3-positive and SSEA3-negative populations with the same retroviral vectors, under identical experimental conditions, and seeded the transduced cells onto inactivated mouse embryonic fibroblasts (MEFs). After three weeks of culture under standard hESC conditions, plates were examined in a double-blind analysis by three independent hESC biologists for iPSC colony formation. Colonies with iPSC morphology were picked and expanded. We observed that all three biological replicates with the transduced SSEA3-negative cells formed many large background colonies (10–27 per replicate, Figure 4A) but no iPSC colonies emerged; in contrast, all three biological replicates with the transduced SSEA3-positive cells resulted in the formation of iPSC colonies (4–5 per replicate, Figure 4B) but very few large background colonies (0–1 per replicate, Table 1). When we further characterized the cell lines derived from the iPSC-like colonies, we observed that they possessed hESC-like morphology, growing as flat colonies with large nucleo-cytoplasmic ratios, defined borders and prominent nucleoli (Figure 4C). When five lines were further expanded and characterized, all demonstrated expression of key pluripotency markers expressed by hESCs, which included: alkaline phosphatase, Nanog, SSEA3, SSEA4, TRA160 and TRA181 (Figure 5A). The SSEA3-selected iPSCs also demonstrated a normal male karyotype (46, XY)(Figure 5B), the ability to differentiate into functional beating cardiomyocytes *in vitro* (Movie S2) and differentiate into representatives of all three germ layers *in vivo* (Figure 5C). Most importantly, since we observed no iPSC colony formation or line derivation from the transduced SSEA3-negative cells, this suggests that these cells possess significantly lower or even no reprogramming potential relative to the SSEA3-expressing cells (Table 1). Additionally, a 10-fold enrichment of primary fibroblasts that strongly express SSEA3 resulted in a significantly greater efficiency (8-fold increase) of iPSC line derivation compared to the control derivation rate (p<0.05, Table 1).

**Figure 6:**
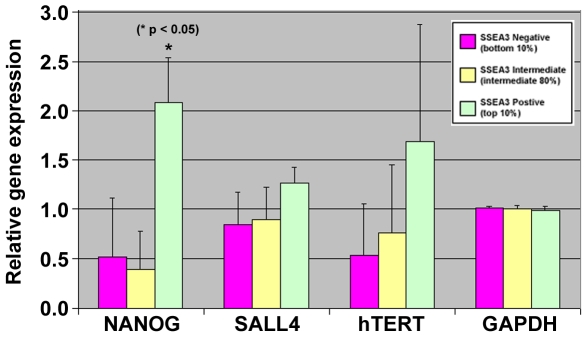
Transcriptional analysis of primary dermal fibroblast subpopulations with differential SSEA3 expression. Relative expression of Nanog, Sall4, hTert and Gapdh from three subpopulations of HUF1 cells: SSEA3-negative cells (representing the bottom 10% for SSEA3 expression/detection), SSEA3 intermediate cells (representing the intermediate 80% of cells between the top and bottom 10% for SSEA3 expression/detection) and SSEA3-positive cells (representing the top 10% for SSEA3 expression/detection). Three biological replicates were analyzed for each sample. The relative gene expression value represents the level of gene expression for each sample compared to the overall average for that gene across the three subpopulations.

**Table 1:** Derivation of human iPSCs from SSEA3 sorted primary dermal fibroblasts.

SSEA3 expression	Biological replicate	iPSC colony formation	iPSC lines derived	Derivation efficiency*
Control (unsorted cells)	1	0	0	N/A
Control (unsorted cells)	2	1	1	N/A
SSEA3-negative cells	1	0	0	0%
SSEA3-negative cells	2	0	0	0%
SSEA3-negative cells	3	0	0	0%
SSEA3-positive cells	1	4	4	800%
SSEA3-positive cells	2	5	4**	800%
SSEA3-positive cells	3	4	4	800%

* Calculated as percentage compared to control derivation.

** HiPS-2E line demonstrated impaired proliferation and thus is not included.

Each biological replicate represented 100,000 transduced cells seeded onto

a 10 cm dish containing MEFs and cultured in hESC media for 3 weeks.

We next examined the expression of genes that might potentially confer the enhanced reprogramming to the SSEA3-positive population, including Nanog [16], Sall4 [17] and hTert [5] as well as the control housingkeeping gene Gapdh. In addition to the SSEA3-positive and negative populations of cells, which represented the top 10% and bottom 10% of SSEA3 expressing cells respectively, we also included the intermediary SSEA3-expressing cells, which represented the remaining 80% of the total HUF1 cell population. Three biological replicates for each of the three subpopulations were analyzed. While no significant differences in gene expression were observed for Sall4, hTert or Gapdh (Figure 6), the analysis revealed that expression of Nanog was significantly increased (p<0.05) in the SSEA3-positive cell population compared to either the SSEA3-intermediate or SSEA3-negative population (Figure 6).

## Discussion

In this study, we unexpectedly observed that SSEA3, a cell surface marker detected on the surface of pluripotent cells, is strongly expressed in a subpopulation of cells derived from a human dermal biopsy. What exactly this subpopulation of cells represents is currently unknown. The SSEA3-positive cells appeared indistinguishable, morphologically, from the SSEA3-negative fibroblasts; furthermore, expression of the SSEA3 antigen is not considered a marker of other cell types such as mesenchymal or epidermal adult stem cells [18], [19]. We hypothesize that SSEA3 expression is either identifying another less differentiated and/or tissue specific stem cell like population that is retrieved with the donor skin biopsy or that with culture, a subpopulation of fibroblasts may acquire the ability to express SSEA3.

Several recent studies have demonstrated that human iPSCs can be generated without permanent integration of genetic factors into the reprogrammed cell chromatin [7], [8], [20]–[22]. While these integration-free human iPSCs avoid the possible oncogenic and insertional mutagenesis issues that prevent the current generation of integration-based iPSCs from being considered for use in human clinical trials [1], the reprogramming efficiency is typically very low. Methods to enhance the reprogramming efficiency would significantly increase the feasibility of this approach, especially for cell types which tend to be more difficult to reprogram, such as the primary adult human fibroblasts used in this study. Our control iPSC derivation efficiency using the HUF1 line was very low, with only 1 iPSC line derived from 200,000 cells. However, in this study we have demonstrated that a 10-fold purification of the top SSEA3-expressing cells could increase the efficiency of reprogramming 8-fold relative to unsorted cells and to a much greater extent relative to the SSEA3-negative cells. This result suggests that further investigation into the identification and isolation of more purified subpopulations from patient-derived somatic cell lines may result in further enhancement of the reprogramming efficiency. In addition to identifying a cell population with enhanced reprogramming efficiency, we also identified an SSEA3-negative population with either significantly reduced reprogramming efficiency or no reprogramming ability. Comparison analysis between the SSEA3-positive and negative populations may help us elucidate the currently poorly understood mechanisms of reprogramming.

Our transcriptional analysis of the SSEA3-positive and negative populations revealed a significantly increased expression of Nanog in the SSEA3-positive population (p<0.05). As increased Nanog expression has been demonstrated to enhance reprogramming efficiency [16], this suggests Nanog may be playing a role in the differential reprogramming observed. However, it should be noted that the level of Nanog expression is thousands of times higher in hESCs and fully reprogrammed iPSCs than in the SSEA3-expressing HUF1 cells, making it likely that other factors may also be playing a role in the differential reprogramming observed. Future studies using global transcriptional and epigenetic profiling should assist in further identifying the differences between the SSEA3-positive and negative subpopulations, and may help elucidate the mechanisms of reprogramming.

In summary, we have reported the identification and isolation of a subpopulation of human dermal fibroblasts that express the pluripotency marker SSEA3, we have demonstrated an enhanced efficiency of generation of iPSCs from these SSEA3-expressing cells and observed no iPSC generation from the non-SSEA3-expressing cells, and we have revealed significantly increased Nanog expression in the SSEA3-expressing fibroblasts, suggesting a possible mechanistic explanation for the differential reprogramming. To our knowledge, this study is the first to identify a pluripotency marker in a heterogeneous population of human dermal fibroblasts, to isolate a subpopulation of cells that have a significantly increased propensity to reprogram to pluripotency and to identify a possible mechanism to explain this differential reprogramming.

## Materials and Methods

### Ethics Statement

Written approval for all somatic derivations and subsequent iPSC generation performed in this study was obtained from the Stanford University Institutional Review Board (IRB protocol 10368) and the Stanford University Stem Cell Research Oversight Committee (SCRO protocol 40), and written informed consent was obtained from each individual participant.

### Isolation of Primary Adult Dermal Human Fibroblast (HUF) Cell Lines

A somatic bank of nine primary adult dermal human fibroblast (HUF) lines were derived and used in this study. The gender, age and disease status/phenotype of the participants were as follows: HUF1 male 28 healthy control, HUF2 male 62 sporadic Parkinson's disease, HUF3 female 30 healthy control, HUF4 male 42 Parkinson's disease (caused by triplication in the α-synuclein gene), HUF5 female 46 (sibling to HUF4), HUF6 female 60 Parkinson's disease (homozygous for G2019S mutation in the leucine-rich repeat kinase 2 gene), HUF7 male 35 Parkinson's disease (heterozygous for G2019S mutation in leucine-rich repeat kinase 2 gene), HUF8 male 45 healthy control and HUF9 female 31 premature ovarian failure. For each HUF line derivation, the adult donor was consented and an inner arm 4 mm skin punch biopsy was obtained at the Stanford University Dermatology Clinic by a qualified dermatologist. The skin biopsies were washed in Ca/Mg-free Dulbecco's Phosphate Buffered Saline (PBS, Invitrogen, Carlsbad, CA, http://www.invitrogen.com) and minced into approximately 12 smaller pieces before being seeded onto gelatin-coated 6-well cell culture plates (Corning Enterprises, Corning, NY, http://www.corning.com) containing mouse embryonic fibroblast (MEF) media consisting of Dulbecco's Modified Eagle Medium (DMEM) supplemented with 10% fetal bovine serum (FBS, Invitrogen) and 100 IU/ml penicillin-streptomycin (Invitrogen), and cultured at 37°C in a 5% CO_2_ incubator. The culture medium was partially changed every two days until biopsy adhesion was observed (usually day 4–6) and then completely changed every two days afterwards. Once the fibroblasts migrated out (usually day 10-12) the attached biopsy fragments and connected epithelial cells were manually removed and the fibroblasts were allowed to expand up to 80–90% confluence. This primary culture was passaged through brief exposure to 0.05% trypsin-EDTA (Invitrogen) and seeded onto gelatin coated 175-cm flasks with fresh culture medium. These somatic cells were cultured until they reached 90% confluence and then frozen down in MEF medium supplemented with 10% dimethyl sulphoxide (DMSO, Sigma-Aldrich, St. Louis, http://www.sigmaaldrich.com).

### Cell Culture

HUF cells were propagated in MEF media consisting of DMEM (Invitrogen) supplemented with 10% FBS (Invitrogen) and 100 IU/ml Penicillin-Streptomycin (Invitrogen). When the cells reached about 80–90% confluence, they were briefly treated with 0.05% trypsin-EDTA (Invitrogen) and split at a 1∶3 ratio into a new dish. Human induced pluripotent stem cells (iPSCs) and H9 human embryonic stem cells (hESCs) were maintained in hESC medium consisting of DMEM/F12 supplemented with 20% Knockout Serum Replacer (KSR, Invitrogen), 2 mM L-glutamine (Invitrogen), 0.1 mM non-essential amino acids (Invitrogen), 0.1 mM β-mercaptoethanol (Millipore, Billerica, MA, http://www.chemicon.com), 100 IU/ml Penicillin-Streptomycin and 10 ng/ml recombinant human basic fibroblast growth factor (β-FGF, Invitrogen). For passaging, individual colonies were simultaneously cut and scraped off from the plate using a customized hockey-style (half-loop) glass pipette tip and transferred to a mitomycin C (Sigma) inactivated MEF seeded dish containing fresh hESC media. All of the research in this study adhered to the National Academy of Sciences guidelines.

### Confocal imaging

Confocal images were collected with a Zeiss LSM510 Meta laser scanning confocal microscope (Carl Zeiss, Jena, Germany, http://www.zeiss.com) with a Zeiss 63′ Plan-Apochromat objective (NA 1.4). For DAPI, excitation was at 405 nm, and a 420–480 nm bandpass filter was used. For Alexa 488, excitation was at 488 nm, and a 505–530 nm bandpass filter was used. Both detector pinholes were set at 1 Airy unit. Sampling was at 0.095 µm/pixel, 12-bits per pixel with a 2.18 µs pixel dwell time.

### SSEA3 live cell staining and FACS cell sorting

Approximately 10 million HUF1 cells were trypsinized through a 5 min exposure to 0.05% trypsin-EDTA (Invitrogen), exposed to MEF media to inactivate the trypsin and then washed twice with ice cold PBS + 2% goat serum (PBS-G). After the first wash the cells were passed through a 40 micrometer filter to remove cellular clumps. For each wash the cells were centrifuged for 5 min at 80 g, the supernatant was removed and the cells were gently resuspended in ice-cold PBS-G. After the washes the cells were resuspended in a 1.5 ml Eppendorf tube in 1 ml of ice-cold PBS-G containing 1∶100 SSEA3 antibody (Millipore, mab4303) and incubated for 45 minutes in the dark at 4°C with gentle rocking. After primary antibody binding the cells were washed three times with ice-cold PBS-G and then resuspended in a 1.5 ml Eppendorf tube in 1 ml of ice-cold PBS-G containing 1∶200 Alexa 488-conjugated goat anti-rat IgM (Invitrogen, A21212) and incubated for 45 minutes in the dark at 4°C with gentle rocking. After secondary antibody binding the cells were washed three times with ice-cold PBS-G and then resuspended in 2 ml of ice-cold PBS-G, passed again through a 40 micrometer filter and then immediately analyzed and sorted on a FACSAria cell sorter (BD Biosciences, San Jose, CA, USA, http://www.bdbiosciences.com) with blue laser excitation (488 nm). Data was analyzed, doublet-exclusion gating was performed and the relevant populations were sorted using BD FACSDiva Software (BD Biosciences). Cells gated within the top 10% for SSEA3 expression were sorted into the “SSEA3-positive” population and cells gated within the bottom 10% for SSEA3 expression were sorted into the “SSEA3-negative” population. Both populations were allowed to adhere, proliferate and recover for 3 days prior to retroviral transduction. Cells used for immunofluorescence analysis were fixed immediately following overnight adherence to remove dead and non-viable cells and cells used for transcriptional analysis were cultured for 6 days prior to analysis.

### Retroviral Production, Infection and iPSC Generation

The following plasmids were obtained from Addgene: pMXs-hOCT3/4 (17217), pMXs-hSOX2 (17218), pMXs-hKLF4 (17219), pMXs-hc-MYC (17220), pUMVC (8449) and pVSV-G (8454) (Addgene Inc., Cambridge, MA, USA, http://www.addgene.org). 293FT cells (Invitrogen) were maintained in MEF media supplemented with 0.5 mg/ml Geneticin (Invitrogen) and cultured until reaching 90–95% confluence before transfection. One day prior to transfection, fresh antibiotic-free culture media was added to the cells. For each 175-cm flask, 293FT cells were transfected with 10 µg of plasmid DNA carrying the transgene (OCT4, SOX2, KLF4 or cMYC) along with 10 µg of the envelope plasmid pVSV-G and 15 µg of the packaging plasmid pUMVC. The transfection was facilitated by 120 µl of Lipofectamine 2000 (Invitrogen) and 15 ml opti-MEM (Invitrogen) for 6 hours and then replaced with 18 ml of fresh MEF medium without antibiotics. After 2 days, the viral supernatant was collected by spinning and passing through a Millex-HV 0.45 um filter unit (Millipore). The viral supernatants were concentrated to 100x by ultracentrifugation (Beckman Coulter, Inc., Fullerton, CA, USA, http://www.beckman.com) at 17,000 RPM for 2.5 hours at 20°C and then resuspended overnight at 4°C in MEF media. These 100x concentrated viral stocks were either used fresh or frozen in aliquots at −80C.

One day before transduction, HUF1 cells were seeded at 10^5^ cells per well of a gelatin coated 6-well plate. On the following day (considered day 0) the concentrated retroviral supernatants were thawed and mixed at a 20x OCT4, 10x SOX2, 10x KLF4, 10x cMYC ratio, supplemented with fresh MEF media up to 2 ml volume (per well) and 8 ng/ml polyprene and then exposed to the HUF1 cells at 37°C and 5% CO^2^. After 24 hours (on day 1) the mixed viral supernatant was removed, the cells were washed twice with PBS and then cultured in fresh MEF medium. On day 2 a second transduction was performed at the same viral concentrations. On day 3 the mixed viral supernatant was again removed, the cells were washed twice with PBS and then cultured in fresh MEF medium. Five days post-transduction (day 5), the cells were resuspended with trypsin, counted and seeded onto 10-cm dishes pre-plated with mitomycin C inactivated MEF feeders. 10^5^ transduced HUF1 cells were seeded per biological replicate. After overnight incubation, the MEF medium was replaced with hESC medium, and thereafter, the medium was changed either every day or every other day, as required. hESC-like colonies started to appear among background colonies around 14 days post-transduction. Large background colonies were classified as colonies with no hESC-like characteristics and a diameter equal or greater than 2.5 mm. Colonies with hESC-like morphology were manually picked and transferred to 12 or 6-well plates pre-plated with mitomycin C inactivated MEF feeders on day 21. Colonies that continued to expand and maintained their hESC-like morphology were further passaged; whereas, those that failed to expand and/or spontaneously differentiated were discarded.

### Alkaline Phosphatase Staining and Immunofluorescence

Alkaline Phosphatase (AP) staining was performed for 30 min at room temperature in the dark using the Vector Red Alkaline Phosphatase Substrate Kit I (Vector Laboratories, Burlingame, CA, http://www.vectorlabs.com), according to the manufacturer's protocol. For immunofluorescence, the cells were fixed in 4% paraformaldehyde/PBS for 20 minutes, washed twice with PBS, and blocked with 4% goat serum in PBS for 30 min, with all procedures performed at room temperature. For Nanog staining, prior to blocking, the cells were permeabilized with 1% Triton-X100 for 1 hour at room temperature. Subsequently, the primary antibodies were added to PBS and incubated overnight at 4°C with gentle shaking. The next day the cells were washed with PBS before fluorescent-conjugated secondary antibodies were added and incubated for an hour at room temperature. Finally, the cells were rinsed with PBS three times and DAPI was used to label the nuclei. Primary antibodies and their dilutions were used as follows: SSEA3 (1∶200, IgM, Millipore, mab4303), SSEA4 (1∶200, IgG, Millipore, mab4304), TRA-1-60 (1∶200, IgM, Millipore, mab4360), TRA-1-81 (1∶200, IgM, Millipore, mab4381), Nanog (1∶100, IgG, Abcam, Cambridge, MA, USA, http://www.abcam.com, ab21603). Secondary antibodies used were: Alexa 594-conjugated goat anti-rat IgM (1∶500, Invitrogen, A21213), Alexa 488-conjugated goat anti-rat IgM (1∶500, Invitrogen, A21212), Alexa 488-conjugated goat anti-mouse IgM (1∶500, Invitrogen, A21042), Alexa 488-conjugated goat anti-mouse IgG (1∶500, Invitrogen, A11001), Alexa 594-conjugated goat anti-rabbit IgG (1∶500, Invitrogen, A11012).

### Karyotyping

Spectral karyotyping (SKY) was performed according to a previously published protocol [23]. Briefly, cells were treated with 0.03 µg/ml KaryoMAX® Colcemid® Solution (Invitrogen) overnight, then treated with 0.05% trypsin (Invitrogen) for 5 minutes at 37°C to re-suspend the cells. The trypsin was inactivated by adding DMEM medium containing 10% FBS. Pre-warmed hypotonic solution containing equal amounts of 0.4% Potassium Chloride and 0.4% Sodium Citrate was slowly added to the cells to enhance swelling at 37°C for 7 minutes. Carnoy's solution (Methanol:Glacial Acetic Acid, 3∶1 ratio) was used to fix the cells for 30 min. The cells were then dropped onto a pre-cleaned slide (Fisher Scientific, Pittsburgh, PA, USA, http://www.fishersci.com) and the metaphase spread quality was determined using a phase-contrast microscope. After a 3–7 days of aging at room temperature, the slide was hybridized with probes from the SkyPaint™ DNA kit for human chromosomes (Applied Spectral Imaging, Vista, CA, USA, http://www.spectral-imaging.com) for 2 days in a 37°C humidified chamber. The finished metaphase spreads were visualized and analyzed using the SkyView spectral imaging system (Applied Spectral Imaging).

### In *Vitro* Differentiation to Beating Cardiomyocytes

For embryoid body formation, iPS cells were seeded into ultra low attachment plates (Corning) containing DMEM + 20% FBS. After 8 days growing in suspension, the cell aggregates were transferred to gelatin-coated dishes containing the same medium to allow the cells to attach. The medium was changed every 2–3 days for up to 3 weeks or until beating cardiomyocytes were observed.

### Teratoma Assay

For each graft, approximately 10^6^ iPS cells were manually harvested, washed and resuspended in a 1.5 ml tube containing 300 µl hESC medium and then injected subcutaneously into female SCID mice (Charles River Laboratories International, Inc., Wilmington, MA, USA, http://www.criver.com). Any visible tumors 4–8 weeks post-transplantation were dissected and fixed overnight with 4% paraformaldehyde/PBS solution. The tissues were then paraffin embedded, sectioned, stained with hematoxylin and eosin, and examined for the presence of tissue representatives of all three germ layers.

### RNA Extraction and Real-time PCR Analysis

Total RNA was purified using RNeasy Mini Kit (Qiagen, Valencia, CA, http://www1.qiagen.com) according to the manufacturer's instructions. 500 ng of RNA was used in reverse transcription with Superscript III (Invitrogen) and random hexamers. 1.25 µl of cDNA from each sample was mixed with master mix consisting of 5 µl Cells Direct 2X reaction mix (Invitrogen), 2.5 µl 0.2X PPP mix (48 genes, Taqman/Applied Biosystems Inc, Foster City, CA, USA, http://www.appliedbiosystems.com), 0.5 µl Platinum Taq (Invitrogen) and 0.75 µl TE Buffer. The reactions were pre-amped using a thermo cycler (Applied Biosystems) under the following conditions: 1 cycle at 95C, 10 minutes and 14 cycles at 95C, 15 seconds and at 60C, 4 minutes. Then the reactions were diluted with TE buffer to a final volume of 20 µl. 2.25 µl of the pre-amplification products were used in the downstream real-time PCR analysis using the Biomark Fluidigm system (Fluidigm Corporation, San Francisco, CA, USA, http://www.fluidigm.com) according to the company's recommendation. The Ct values for each sample and gene were normalized relative to GAPDH, RPLPO and CTNNB1 by qBasePlus (Biogazelle, Zulte, Belgium, http://www.biogazelle.com). The level of gene expression for each sample was compared to the overall average for that gene, across the three different HUF1 subpopulations (SSEA3-negative, SSEA3-intermediate and SSEA3-positive) to produce a relative gene expression value.

### Statistical analysis

Analysis of variance (ANOVA) statistical comparisons were performed using Statview Software (SAS Institute, Inc., Cary, NC, USA, http://www.jmp.com) with statistical significance set at 0.05.

## Supporting Information

Movie S1 Cardiomyocytes derived from HiPS1-control EBs. Beating cardiomyocytes observed following spontanous differentiation of HiPS1-control embryoid bodies (EBs)
(1.32 MB MOV)

Movie S2 Cardiomyocytes derived from SSEA3-selected HiPS-2C EBs. Beating cardiomyocytes observed following spontanous differentiation of SSEA3 selected HiPS-2C embryoid bodies (EBs)
(2.50 MB MOV)
